# Molecular profiling of cetuximab and bevacizumab treatment of colorectal tumours reveals perturbations in metabolic and hypoxic response pathways

**DOI:** 10.18632/oncotarget.6241

**Published:** 2015-10-26

**Authors:** David W. Greening, Sze Ting Lee, Hong Ji, Richard J. Simpson, Angela Rigopoulos, Carmel Murone, Catherine Fang, Sylvia Gong, Graeme O'Keefe, Andrew M. Scott

**Affiliations:** ^1^ Department of Biochemistry and Genetics, La Trobe Institute for Molecular Science, La Trobe University, Melbourne, Australia; ^2^ Department of Molecular Imaging and Therapy, University of Melbourne, Austin Hospital, Melbourne, Australia; ^3^ Tumour Targeting Laboratory, Olivia Newton-John Cancer Research Institute, Melbourne, Australia; ^4^ School of Cancer Medicine, La Trobe University, Melbourne, Australia

**Keywords:** cetuximab, bevacizumab, cancer therapeutics, metabolism, hypoxia

## Abstract

Angiogenesis and epidermal growth factor receptor (EGFR) inhibition has been shown to have anti-tumour efficacy, and enhance the therapeutic effects of cytotoxic chemotherapy in metastatic colorectal cancer. The interplay of signalling alterations and changes in metabolism and hypoxia in tumours following anti-VEGF and anti-EGFR treatment is not well understood. We aimed to explore the pharmacodynamics of cetuximab and bevacizumab treatment in human colon carcinoma tumour cells *in vitro* and xenograft models through proteomic profiling, molecular imaging of metabolism and hypoxia, and evaluation of therapy-induced changes in tumour cells and the tumour microenvironment. Both cetuximab and bevacizumab inhibited tumour growth *in vivo*, and this effect was associated with selectively perturbed glucose metabolism and reduced hypoxic volumes based on PET/MRI imaging. Global proteomic profiling of xenograft tumours (in presence of cetuximab, bevacizumab, and combination treatments) revealed alterations in proteins involved in glucose, lipid and fatty acid metabolism (e.g., GPD2, ATP5B, STAT3, FASN), as well as hypoxic regulators and vasculogenesis (e.g., ATP5B, THBS1, HSPG2). These findings correlated with western immunoblotting (xenograft lysates) and histological examination by immunohistochemistry. These results define important mechanistic insight into the dynamic changes in metabolic and hypoxic response pathways in colorectal tumours following treatment with cetuximab and bevacizumab, and highlight the ability of these therapies to selectively impact on tumour cells and extracellular microenvironment.

## INTRODUCTION

Colorectal cancer (CRC) is the third most common cancer and the third leading cause of cancer death in men and women worldwide, with nearly 1.4 million new cases diagnosed in 2012, representing 9.7% of cancers worldwide [1]. CRC continues to be a significant public issue with >500,000 deaths worldwide attributed to this disease annually. About 25% of patients present with metastatic disease, and of this group, 50-75% will have the disease confined to the liver [2-5]. Early stage detection significantly improves the clinical outcome [6] although this is often made difficult by the lack of specific symptoms [7]. With over 70% of CRC cases detected at advanced stages, screening remains unsatisfactory and non-specific [8]. Further, in patients who present initially with early-stage disease, up to 50% will eventually develop metastatic disease.

Significant advances have been made in the treatment of metastatic colorectal cancer (mCRC) [9]. Refinements to cytotoxic chemotherapy regimens have incrementally improved median life expectancy in patients with mCRC, however, such gains have often been attributed with increased toxicity. Recent treatment advances have recognized the role of monoclonal antibodies in the management of mCRC [10]. International guidelines recommend combination chemotherapy with the addition of a monoclonal antibody (i.e., bevacizumab) for the first-line treatment of mCRC, while for chemoresistant mCRC, cetuximab (erbitux) or panitumumab (vectibix) are recommended as treatment of patients with wild-type *K-Ras* tumours. These targeted agents, now validated in mCRC and other tumour types, target and perturb critical cell-signalling pathways that regulate (stimulate) tumour angiogenesis and growth.

Bevacizumab disrupts angiogenesis by binding to vascular endothelial growth factor (VEGF)-A, reducing availability of this ligand to its receptors, and preventing their activation. Based on pre-clinical models, bevacizumab has been shown to have both anti-vascular and anti-angiogenic effects, resulting in regression of existing tumour vasculature [11-13], and inhibition of new and recurrent tumour vessel growth [14, 15], respectively. This results in a synergistic reduction in tumour size and inhibition in tumour growth. Bevacizumab remains the most important and well-studied drug among the known anti-angiogenic agents. Bevacizumab was the first agent to affect survival in patients with mCRC, improving survival by 30% [16], with more recent phases II and III clinical trials further demonstrating its significant beneficial effect [17].

Cetuximab, a monoclonal antibody, binds to the extracellular domain of the epidermal growth factor receptor (EGFR), which is overexpressed and active in mCRC. EGFR, a receptor for EGF present on the surface of normal epithelium, is overexpressed in up to 80% of colorectal tumours [18, 19]. EGFR mediates cell differentiation, proliferation, migration, angiogenesis and apoptosis, all of which are deregulated in mCRC [20]. Cetuximab directly inhibits tumour growth, induction of apoptosis, inhibition of angiogenesis, inhibition of metastasis, and also exerts anti-angiogenic effects by blocking ligand-induced phosphorylation of EGFR on endothelial cells [20, 21]. EGF blockade also interferes with VEGF production by tumour cells, suggesting a complementary anti-tumour effect during combination therapies of cetuximab and bevacizumab [22, 23]. Interestingly, tumours with mutations in *K-Ras* are associated with resistance to cetuximab therapy [24, 25]. This underscores the need to identify factors that can predict response or resistance to cetuximab or other such targeted therapies [25].

To explore the pharmacodynamics of bevacizumab and cetuximab treatment, and further our understanding of therapy-induced changes in tumour cells and the tumour microenvironment, we profiled different human-derived CRC xenografts following treatment with these agents alone or in combination. The human CRC cell lines HT-29 and LIM1215 were utilised to investigate responsiveness to bevacizumab/cetuximab monotherapy or combination-based treatment using human xenograft models. Most significantly, we demonstrate that these targeted therapies had a selective effect on different tumour cells (resistant/weakly responsive), resulting in changes in tumour growth, metabolism, signalling, vasculature, and tissue oxygenation. These findings contribute towards understanding the underlying therapeutic and biological processes associated with monoclonal therapy of EGFR and VEGF anti-tumour activities, and identify further potential protein markers that may contribute in assessment of mCRC treatment response.

## RESULTS AND DISCUSSION

Cetuximab and bevacizumab are both approved for the treatment of mCRC, however the mechanism of action of both drugs and impact on signalling and metabolic changes in both tumour cells and microenvironment are not fully understood. We therefore explored the effects of treatment of different human CRC models, HT-29 and LIM1215, grown as xenografts with cetuximab and bevacizumab (in addition to combination treatment). To directly monitor and characterise specific anti-tumour activity, molecular imaging of metabolism and hypoxia, proteomic profiling and immunohistochemistry were employed.

### Treatment of colorectal xenografts with cetuximab and bevacizumab

**Figure 1:**
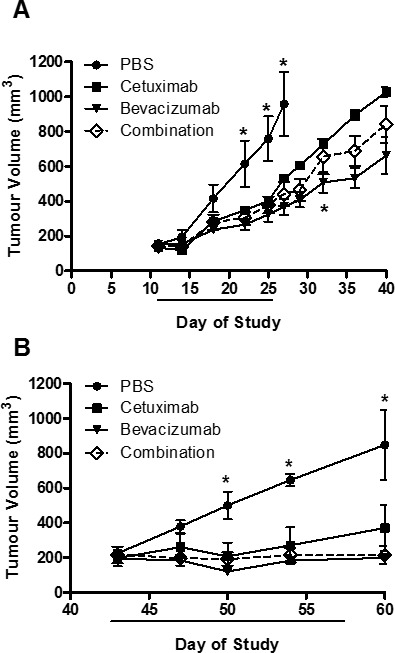
HT-29 and LIM1215 tumour xenograft treatment **A.** HT-29 (2×10^6^ cells/site) and **B.** LIM1215 (5×10^6^ cells/site) were injected subcutaneously into BALB/c mice inguinal regions. Tumour volumes were measured at indicated times (*n* = 5; mean tumour volume ± SEM; *P < 0.05). Treatment cohorts included (1) vehicle (PBS) 2x/week; (2) cetuximab −20 mg/kg 2x/week; (3) bevacizumab −10 mg/kg 2x/week; or (4) the combination treatment both cetuximab (20 mg/kg) and bevacizumab (10 mg/kg) 2x/week.

To explore the effects of monotherapy as well as combined therapeutic effect, we subcutaneously injected LIM1215 or HT-29 cells into BALB/c mice, and monitored tumour growth following treatment with control (vehicle, PBS), cetuximab, bevacizumab or cetuximab and bevacizumab combined. Cetuximab and bevacizumab significantly inhibited HT-29 tumour growth compared to control, although the combination of cetuximab and bevacizumab did not provide additional therapeutic benefit (Figure 1A). In LIM1215 tumours, the significant growth inhibition observed with cetuximab and bevacizumab treatment was more pronounced compared to HT-29 tumours, and combination treatment also did not demonstrate a significantly improved effect (Figure 1B). These results are consistent with the known differences in response of mCRC tumours to these therapeutics, and highlight the therapeutic activity of EGFR and VEGF-A antibodies in CRC.

### Glucose metabolism and hypoxia following treatment

**Figure 2:**
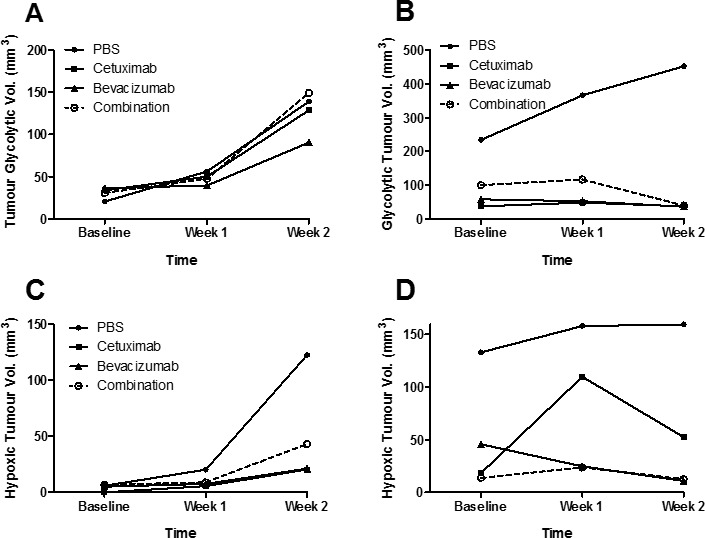
Glycolytic and hypoxic tumour volumes following treatment with cetuximab and bevacizumab ^18^F-FDG tumour glycolytic volumes of **A.** HT-29 and **B.** LIM1215 tumours following treatment with PBS, cetuximab, bevacizumab, or combination. ^18^FMISO PET hypoxic tumour volumes of **C.** HT-29 and **D.** LIM1215 tumours following treatment with PBS, cetuximab, bevacizumab, or combination.

To evaluate the *in vivo* metabolism of tumours following treatment, PET/MRI imaging of mice with HT-29 and LIM1215 tumours at baseline and following treatment was performed with ^18^F-FDG PET, and ^18^F-FMISO PET (Supplementary Figure 1). No difference in glycolytic volumes were found between treated and control HT-29 tumours (Figure 2A), although reduced glycolytic volumes were observed for treated LIM1215 tumours (Figure 2B). Both HT-29 (Figure 2C) and LIM1215 tumours showed reduced hypoxic volumes following treatment with cetuximab and bevacizumab, more marked in the LIM1215 treated tumours. The reduction in hypoxia reflects the effects of cetuximab and bevacizumab on vasculature through both direct and paracrine effects [25-27].

### Immunohistochemistry analysis of tumours

**Figure 3:**
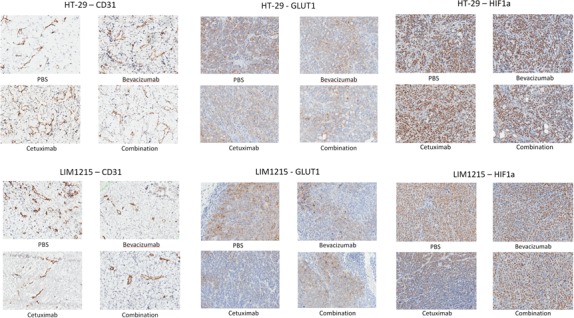
Expression of CD31, GLUT-1 and HIF1-alpha following treatment Immunohistochemistry results of CD31, GLUT-1 and HIF-1alpha in HT-29 and LIM1215 tumours following treatment with PBS, bevacizumab, cetuximab, or combination.

GLUT-1 expression was reduced following treatment by both bevacizumab and cetuximab, and combination treatment, in both HT-29 and LIM1215 tumours, with cetuximab effect most markedly observed in LIM1215 tumours (Figure 3). HIF-1alpha expression was slightly reduced following treatment with bevacizumab and cetuximab, and combination in HT-29 tumours, however, a greater reduction was observed in LIM1215 tumours (Figure 3), consistent with the hypoxic volume reduction following treatment seen on ^18^F-FMISO PET (Figure 2C and 2D). CD31 expression, a vascular endothelial marker consistent with significant vascularisation, showed minimal change following treatment with bevacizumab or cetuximab in HT-29 tumours, but more marked reduction was seen in LIM1215 tumours (Figure 3), also consistent with the HIF-1alpha results. EGFR expression was reduced following cetuximab treatment in HT-29 and LIM1215 tumours, and VEGF-A showed minimal change following treatment with bevacizumab or cetuximab in either HT-29 or LIM1215 tumours (data not shown). These findings correlate with the molecular imaging studies and identify key proteins regulated by cetuximab and bevacizumab treatment.

### Proteome analysis of tumour xenograft-derived lysates in response to drug treatments

**Figure 4:**
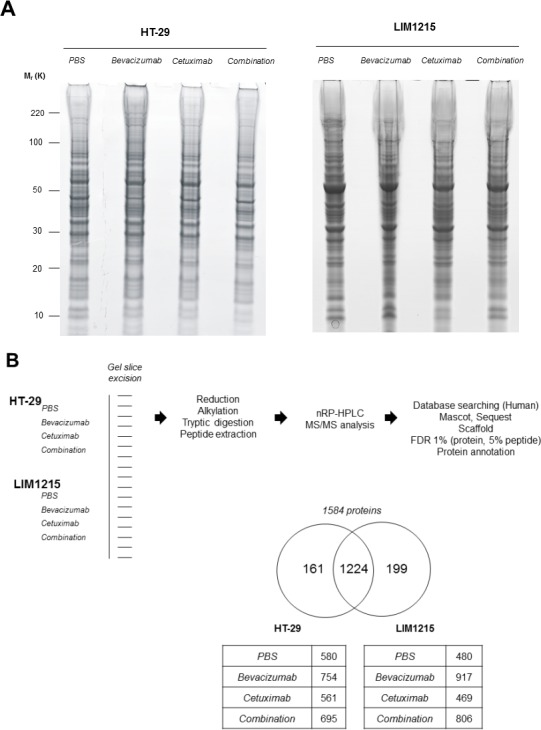
Proteomic characterisation of HT-29 and LIM1215 tumour xenografts in response to cetuximab and/or bevacizumab treatment **A.** Proteins extracted from cetuximab and/or bevacizumab treated HT-29 and LIM1215 tumours were separated by 1D-SDS-PAGE and stained with Imperial™ Protein Stain (10 μg). **B.** Individual gel slices were excised and subjected to in-gel reduction, alkylation, and tryptic digestion. Extracted peptides were separated by nRP-HPLC followed by data-dependent mass spectrometry analysis, database searching, stringent peptide/protein identification, bioinformatic analyses, and protein annotation. A two-way Venn diagram of bevacizumab and cetuximab treated tumour lysates is shown, with 1224 proteins commonly identified between each cell line. The number of identified proteins for control, cetuximab, bevacizumab, and combination treatment cohorts are shown for each tumour-derived xenograft (Supplementary Table S1).

To gain insights into the tumour protein expression changes following treatment with bevacizumab and cetuximab we extracted xenograft-derived proteins and performed in-depth proteome profiling using GeLC-MS-MS. Protein visualisation using Imperial™ Protein Stain indicates differences in HT-29 and LIM1215 tumour-derived protein profiles in response to bevacizumab and cetuximab, and combination treatments (Figure 4A). GeLC-MS/MS profiling identified a total of 1,584 proteins, comprising 1385 and 1423 in HT-29 and LIM1215, respectively (Figure 4B and Supplementary Table S1). For HT-29 xenograft lysates, we identified 580 (control), 754 (bevacizumab), 561 (cetuximab), and 695 (combination) proteins, while for LIM1215 xenograft lysates we identified 480 (control), 917 (bevacizumab), 469 (cetuximab), and 806 (combination) proteins. To indicate differential protein expression between HT-29 and LIM1215 xenograft samples, and different therapeutic treatments we used normalised relative spectral count ratios (Rsc) to correlate with fold-change [28, 29, 30] and Western immunoblotting.

### Anti-tumour effects of cetuximab and bevacizumab mediated through altered cellular metabolism

**Figure 5:**
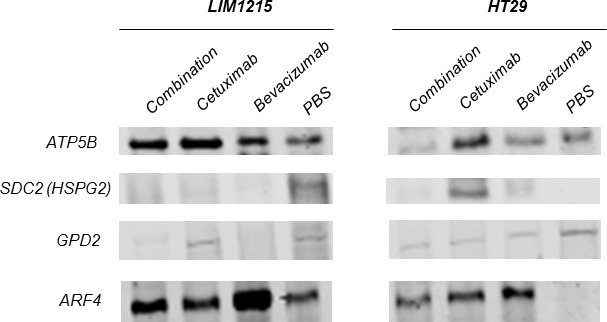
Validation of anti-tumour effects of cetuximab and bevacizumab mediated through altered cellular metabolism Proteins were extracted from tumour xenografts for each treatment cohort (independent from the tumour xenograft lysates performed for proteomic profiling), obtained from pooled tumour xenograft samples from the validation experimental group (*n* = 3). Immunoblotting analysis of the expression of ATP5B, SDC2, GPD2, ARF4 in both LIM1215 and HT-29 tumour xenograft lysates was performed (*n* = 3; pooled for each treatment cohort, independent biological replicates performed for each antibody).

In response to cetuximab and bevacizumab, significant changes in tumour xenograft protein expression were observed associated with cellular and glycolytic metabolic processing (Table 1). Most notably, glycerol-3-phosphate dehydrogenase (GPD2, Rsc −16.6) and ATP Synthase (ATP5B, Rsc −25.7) (Figure 5) were significantly reduced in expression following bevacizumab treatment in LIM1215 and HT-29, respectively. Further, glucose and glycolytic metabolic-related proteins including STAT3, G6PD PGM1, ILF3, PC, and THBS1 were also significantly reduced in expression following bevacizumab treatment in both LIM1215 and HT-29 tumours.

**Table 1:** Relative quantification by label-free spectral counting of proteins associated with cellular metabolism

A - Cetuximab			HT-29^b^			LIM1215^b^			
Protein Acc^a^	Gene Name^a^	Protein Description^a^	Rsc (PBS v bevacizumab)	Rsc (PBS v cetuximab)	Rsc (PBS v Combination)	Rsc (PBS v bevacizumab)	Rsc (PBS v cetuximab)	Rsc (PBS v Combination)	Associated function^a^
P11216	PYGB	Glycogen phosphorylase, brain form	−16.5	1.2	−14.2	−3.8	−3.5	−3.5	glucose metabolic process; glycogen catabolic process; glycogen phosphorylase activity
Q96PK6	RBM14	RNA-binding protein 14	−1.5	1.1	−1.3	−3.8	−3.5	−3.5	DNA recombination; DNA repair
P42704	LRPPRC	Leucine-rich PPR motif-containing protein, mitochondrial	−10.6	1.1	−9.1	−23.8	−4.3	−21.7	mitochondrion transport along microtubule; mRNA transport; regulation of transcription
Q9NYU2	UGGT1	UDP-glucose:glycoprotein glucosyltransferase 1	−4.1	−1.1	−3.5	−5.7	−5.2	−5.1	cellular protein metabolic process; glucosyltransferase activity
Q01813	PFKP	6-phosphofructokinase type C	−8.6	−1.2	−7.4	−5.7	−5.2	−5.1	glucose metabolic process; glycolytic process
Q00341	HDLBP	Vigilin	−11.3	−1.6	−9.7	−29.3	−5.3	−26.7	cholesterol metabolic process; lipid transport
P05023	ATP1A1	Sodium/potassium-transporting ATPase subunit alpha-1	−15.2	2.7	−13.0	−10.2	−9.3	−9.3	cellular potassium ion homeostasis
Q06210	GFPT1	Glutamine--fructose-6-phosphate aminotransferase	−4.7	2.7	−4.1	−12.0	−10.9	−10.9	cellular protein metabolic process; energy reserve metabolic process
P53396	ACLY	ATP-citrate synthase	−3.4	−2.2	−2.9	−13.8	−12.6	−12.6	cellular carbohydrate metabolic process; cellular lipid metabolic process; lipid biosynthetic process; positive regulation of cellular metabolic process
P49327	FASN	Fatty acid synthase	−11.9	1.4	−10.2	−20.2	−18.4	−18.4	cellular lipid metabolic process; energy reserve metabolic process; fatty acid biosynthetic process; fatty acid metabolic process
P11940	PABPC1	Polyadenylate-binding protein 1	−6.0	−1.2	−5.2	−12.0	−10.9	−10.9	cellular protein metabolic process
P11498	PC	Pyruvate carboxylase, mitochondrial	−1.5	−1.7	−1.3	−13.8	−12.6	−12.6	gluconeogenesis; glucose metabolic process; lipid metabolic process
**B - Bevacizumab**									
P40763	STAT3	Signal transducer and activator of transcription 3	1.2	1.1	1.4	−4.8	−2.4	−4.3	cell proliferation; cell differentiation
P11413	G6PD	Glucose-6-phosphate 1-dehydrogenase	−2.8	1.6	−2.4	−5.7	−2.0	−5.1	carbohydrate metabolic process; lipid metabolic process
P36871	PGM1	Phosphoglucomutase-1	−1.5	3.5	−1.3	−6.6	−1.4	−6.0	gluconeogenesis; glucose metabolic process
Q12906	ILF3	Interleukin enhancer-binding factor 3	−2.8	1.8	−2.4	−6.6	−3.3	−6.0	viral defense; transcription
P11498	PC	Pyruvate carboxylase, mitochondrial	−1.5	−1.7	−1.3	−13.8	−12.6	−12.6	gluconeogenesis; glucose metabolic process; lipid metabolic process
P06576	ATP5B	ATP synthase subunit beta	−25.7	−1.7	−8.5	10.7	11.5	13.3	angiogenesis; cellular metabolic process; generation of precursor metabolites and energy; lipid metabolic process
P43304	GPD2	Glycerol-3-phosphate dehydrogenase	−2.8	1.3	−2.4	−16.6	−1.0	−15.1	cellular lipid metabolic process; gluconeogenesis

a Protein description, Gene name, UniProt acc, and molecular function annotated from UniProt (http://www.uniprot.org/)

b Normalised spectral count ratio (Rsc) between datasets

Citrate synthase (CS, Rsc -1.2) and fatty acid synthase (FASN, Rsc -18.4) were reduced following cetuximab treatment of HT-29 and LIM1215 tumour xenografts, respectively (Table 1). Further, glucose and glycolytic metabolic-related proteins including PYGB, RBM14, LRPPRC, UGGT1, PFKP, HDLBP, ATP1A1, GFPT1, PABPC1, and PC were also significantly reduced in expression following cetuximab treatment in LIM1215 tumours. These findings are consistent with deregulated tumour metabolism (glycolysis, gluconeogenesis, TCA cycle, lipid synthesis) following bevacizumab and cetuximab treatment, particularly in the more responsive LIM1215 treated tumours. This also aligns with the reduced FDG PET uptake (Figure 2A), and immunohistochemistry (Figure 3), and suggests that the metabolic changes are not fully explained by changes in glucose transporter expression.

With regards to alterations in EGFR following cetuximab treatment, several proteins were enriched independently in LIM1215 (and not HT-29) following cetuximab treatment, and were associated with cellular metabolism and signal transduction (Table 2). This selective deregulation is possibly attributed to a reduced response to cetuximab treatment in HT-29 tumours (based on resistance/reduced sensitivity [31]), although some downregulation of total EGFR following treatment was shown (Figure 3). Increased expression of ATP5B (Rsc 10.7), LDHA (Rsc 21.1), CS (Rsc 4.9), the ADP-ribosylation factors (ARF) 3/4/5 (Rsc 7.2-11.9) (Figure 5) and RAB1A/B (Rsc 5.6-8.8) were observed. Interestingly, ARF4 is known to interact with EGFR and mediate the EGF-dependent signal pathway [32]. ARF4 is a critical molecule that directly regulates cellular PLD2 activity, and that this ARF4-mediated PLD2 activation stimulates AP-1-dependent transcription in the EGF-induced cellular response [33]. Interestingly, this was confirmed using IHC with reduced total EGFR following cetuximab treatment (data not shown). Further upregulated protein classes included cytoskeleton-related proteins (TUBB4B, TUBB, ACTR3), and the protein ENO1 (Rsc 7.9), associated with glycolysis, gluconeogenesis, TCA cycle, and lipid synthesis. This selective deregulation of various metabolic-associated proteins is in agreement with increased anaerobic metabolism as a prominent feature observed in a syngenic model of acquired resistance to anti-EGFR therapy in CRC [34].

**Table 2:** Relative quantification by label-free spectral counting of cetuximab-resistant protein expression

			HT-29^b^			LIM1215^b^			
Protein Acc^a^	Gene Name^a^	Protein Description^a^	Rsc (PBS v bevacizumab)	Rsc (PBS v cetuximab)	Rsc (PBS v Combination)	Rsc (PBS v bevacizumab)	Rsc (PBS v cetuximab)	Rsc (PBS v Combination)	Associated function^a^
P68371	TUBB4B	Tubulin beta-4B chain	−2.2	−1.0	1.2	48.1	28.3	52.1	cytoskeleton remodelling, cytotoxicity
P07437	TUBB	Tubulin beta chain (Tubulin beta-5 chain)	−2.6	1.2	1.1	31.9	16.6	36.5	cytoskeleton remodelling, cytotoxicity
P00338	LDHA	L-lactate dehydrogenase A chain	2.7	2.8	5.0	21.1	12.2	26.7	Cellular metabolic process, glycolytic process
P61204	ARF3	ADP-ribosylation factor 3	15.0	1.1	10.6	8.0	11.9	4.9	Cellular metabolic process, glycolytic process
P06576	ATP5B	ATP synthase subunit beta, mitochondrial	−25.7	−1.7	−8.5	10.7	11.5	13.3	Cellular metabolic process, generation of precursor metabolites and energy
P62820	RAB1A	Ras-related protein Rab-1A	16.0	1.1	19.9	8.7	8.8	12.7	Signal transduction
P06733	ENO1	Alpha-enolase	−3.0	1.1	−2.3	20.7	7.9	22.8	Glycolytic process
P84085	ARF5	ADP-ribosylation factor 5	11.1	1.1	8.3	6.6	7.2	5.6	Signal transduction
P18085	ARF4	ADP-ribosylation factor 4	5.1	1.5	4.0	7.3	7.2	8.8	Signal transduction, apoptotic process
P31946	YWHAB	14-3-3 protein beta/alpha	34.9	1.1	33.8	30.1	5.6	29.2	Apoptotic process
P62258	YWHAE	14-3-3 protein epsilon	32.9	1.9	28.0	27.2	5.6	29.2	Apoptotic process
P27348	YWHAQ	14-3-3 protein theta	22.0	1.1	28.0	25.1	5.6	25.2	Apoptotic process
P50454	SERPINH1	Serpin H1	−9.9	−2.3	−8.5	18.7	5.6	9.5	regulation of proteolysis
Q9H0U4	RAB1B	Ras-related protein Rab-1B	16.0	1.1	16.4	8.0	5.6	11.1	Signal transduction
O00303	EIF3F	Eukaryotic translation initiation factor 3 subunit F	−4.1	−2.6	−3.5	6.6	5.6	8.8	regulation of translational initiation
P31947	SFN	14-3-3 protein sigma	8.2	−3.2	9.9	25.8	4.9	26.8	Apoptotic process
P31943	HNRNPH1	Heterogeneous nuclear ribonucleoprotein H	−1.3	−2.2	1.4	11.5	4.9	12.7	regulation of RNA splicing
O75390	CS	Citrate synthase, mitochondrial	−3.4	−1.2	1.4	8.7	4.9	8.0	cellular carbohydrate metabolic process
P61158	ACTR3	Actin-related protein 3	−5.4	−1.5	−4.6	7.3	4.9	7.2	cytoskeleton remodelling
									
P49327	FASN	Fatty acid synthase	−11.9	1.4	−10.2	−20.2	−18.4	−18.4	Cellular metabolic process, lipid metabolism
Q08211	DHX9	ATP-dependent RNA helicase A	−5.4	1.3	−4.6	−16.6	−15.1	−15.1	ATP catabolic process
P11940	PABPC1	Polyadenylate-binding protein 1	−6.0	−1.2	−5.2	−12.0	−10.9	−10.9	cellular protein metabolic process
P35241	RDX	Radixin	−14.5	1.0	−12.5	−11.1	−10.1	−10.1	cytoskeleton remodelling
P07384	CAPN1	Calpain-1 catalytic subunit	−4.7	1.4	−4.1	−8.4	−7.6	−7.6	regulation of cell proliferation
P39656	DDOST	Dolichyl-diphosphooligosaccharide--protein glycosyltransferase 48 kDa subunit	1.2	1.1	3.7	−1.5	−6.8	−6.8	Cellular metabolic process, glycolytic process
Q99613	EIF3C	Eukaryotic translation initiation factor 3 subunit C	1.2	1.1	2.6	−7.5	−6.8	−6.8	cellular protein metabolic process
Q14152	EIF3A	Eukaryotic translation initiation factor 3 subunit A	−8.0	1.1	−6.9	−17.5	−6.1	−15.9	cellular protein metabolic process
E7EX73	EIF4G1	Eukaryotic translation initiation factor 4 gamma 1	−5.4	−1.1	−4.6	−6.6	−6.0	−6.0	cellular protein metabolic process
Q9NYU2	UGGT1	UDP-glucose:glycoprotein glucosyltransferase 1	−4.1	−1.1	−3.5	−5.7	−5.2	−5.1	Cellular metabolic process, glycolytic process
Q01813	PFKP	6-phosphofructokinase type C	−8.6	−1.2	−7.4	−5.7	−5.2	−5.1	Cellular metabolic process, glycolytic process

a Protein description, Gene name, UniProt acc, and molecular function annotated from UniProt (http://www.uniprot.org/)

b Normalised spectral count ratio (Rsc) between datasets

For proteins down-regulated independently in LIM1215 (and not HT-29) in response to cetuximab treatment, various cellular metabolic-associated and glycolytic-associated proteins were found (Table 2). These included proteins associated with cellular and lipid metabolism (FASN (Rsc -18.4)), cellular protein metabolism (PABPC1 (Rsc -10.9), EIF3A/C (Rsc -6.1-6.8), EIF4G1 (Rsc -6.0)), and cellular metabolism/glycolytic processing (DDOST (Rsc -6.8), UGGT1 (Rsc -5.2), PFKP (Rsc -5.2)). Recently, EGFR has been shown to promote glucose metabolism of chondrosarcoma cells through the upregulation of glycolytic enzymes [35]. Interestingly, cisplatin-resistant chondrosarcoma cells showed upregulated glucose metabolism and EGFR signalling pathway. With regards to FASN expression in response to cetuximab, FASN has been linked to acquired docetaxel/trastuzumab/adriamycin resistance in breast cancer or intrinsic gemcitabine and chemo- or radiotherapy resistance in pancreatic cancer. FASN expression is significantly upregulated in pancreatic cancer [36] and inhibition of FASN by siRNA or the FAS inhibitor orlistat reduces gemcitabine resistance, whereas ectopic overexpression of FASN contributes to intrinsic resistance to gemcitabine and radiation [37, 38]. FASN-induced radiation resistance may result from decrease in radiation-mediated ceramide production, leading to reduced caspase 8-induced apoptosis. However, the mechanism of FASN-induced gemcitabine resistance remains to be elucidated [39]. FASN expression in normal adult tissues is generally very low or undetectable, and it is significantly upregulated and correlates with poor prognosis in many types of cancer. The metabolic products of the FASN complex are rapidly consumed by actively dividing cells and recent data demonstrates that FASN expression is important for tumour growth and survival, suggesting that FASN is a metabolic oncogene [40].

### Anti-tumour effects of cetuximab and bevacizumab mediated through altered angiogenesis and hypoxia

In agreement with Figure 2C and 2D, we further identified significant deregulation in comparison to vehicle in protein expression associated with HT-29 and LIM1215 tumour xenograft treatments associated with altered angiogenesis and hypoxia. With respect to angiogenesis (Table 3), we observed upregulation in response to cetuximab and bevacizumab (and combination) of 11 proteins in LIM1215 including ATP5B (Rsc 11.5/10.7), ENO1 (Rsc 7.9/20.7) in both cetuximab and bevacizumab (and combination), TGFBI (Rsc 4.9), STAT1 (Rsc 4.0), ANPEP (Rsc 3.3), PTPN6 (Rsc 2.5), and TYMP (Rsc 2.5) in cetuximab, and LDHB (Rsc 12.2), IDH2 (Rsc 10.1) in bevacizumab. Although we observed only a marginal therapeutic effect with HT-29 in response to cetuximab and bevacizumab, we report that HSPG2 (Rsc 6.2/4.2) was upregulated in response to cetuximab and bevacizumab (Figure 5), and THBS1 (Rsc 12.2) in response to bevacizumab. Interestingly, cetuximab treatment reduced HSPG expression (Rsc -3.8) in LIM1215 (Figure 5), which is involved in angiogenesis and cell-cell interactions. Therefore, the protein expression changes in THBS1 and HSPG align with altered vasculogenesis and cell-cell interactions.

**Table 3:** Angiogenic-associated proteins identified in tumour xenografts in response to anti-tumour treatments

			HT-29^b^			LIM1215^b^		
Protein Acc^a^	Gene Name^a^	Protein Description^a^	Rsc (PBS v bevacizumab)	Rsc (PBS v cetuximab)	Rsc (PBS v Combination)	Rsc (PBS v bevacizumab)	Rsc (PBS v cetuximab)	Rsc (PBS v Combination)
P06576	ATP5B	ATP synthase subunit beta, mitochondrial	−25.7	−1.7	−8.5	10.7	11.5	13.3
P06733	ENO1	Alpha-enolase	−3.0	1.1	−2.3	20.7	7.9	22.8
Q15582	TGFBI	Transforming growth factor-beta-induced protein ig-h3	−1.5	1.1	−1.3	−1.1	4.9	−1.0
P42224	STAT1	Signal transducer and activator of transcription 1-alpha/beta	1.2	1.1	1.4	−2.0	4.0	−1.9
P15144	ANPEP	Aminopeptidase N (AP-N)	−9.3	−1.6	−8.0	−1.1	3.3	−1.0
P29350	PTPN6	Tyrosine-protein phosphatase non-receptor type 6	1.2	1.1	1.4	−1.1	2.5	−1.0
P19971	TYMP	Thymidine phosphorylase	1.2	1.1	1.4	−1.1	2.5	5.6
P07195	LDHB	L-lactate dehydrogenase B chain	3.2	2.6	3.0	12.2	−1.0	11.1
P48735	IDH2	Isocitrate dehydrogenase [NADP], mitochondrial	−8.6	−1.1	1.5	10.1	−1.0	6.4
P98160	HSPG2	Basement membrane-specific heparan sulfate proteoglycan core protein	4.2	6.2	1.4	−3.8	−3.5	−3.5
P07996	THBS1	Thrombospondin-1	1.2	12.2	1.4	−15.6	−1.7	−14.2

a Protein description, Gene name, UniProt acc, and molecular function annotated from UniProt (http://www.uniprot.org/)

b Normalised spectral count ratio (Rsc) between datasets

Hypoxia is recognized as an important factor contributing to cancer development and drug resistance [41-43]. With regards to hypoxia associated proteins expression changes (based on increased tumour hypoxia volume associated with HT-29, Figure 2C), we report THBS1 (Rsc 12.2), SLC25A13 (Rsc 3.0), GFPT1 (Rsc 2.7), and NPEPPS (Rsc 2.4) were selectively upregulated in response to cetuximab (Table 4). It has been shown that hypoxia induced drug resistance occurs in gastric cancer cells, whereby cetuximab enhanced oxaliplatin-induced cytotoxicity and apoptosis in normoxia and caused a reversal of drug resistance in hypoxia [44]. Cetuximab was shown to inhibit HIF-1α expression via the MAPK/ERK and PI3K/AKT signalling pathways and functions to overcome drug resistance induced by hypoxia. In response to bevacizumab, PDLIM1 (Rsc 9.1), FABP1 (Rsc 7.1), PSMA7 (Rsc 6.2), TCEB2 (Rsc 6.2), and ACAA2 (Rsc 5.2) were upregulated in HT-29 xenograft tumours (Table 4). These data further support the IHC results (Figure 3) of slightly reduced expression of HIF-1alpha, and reduced hypoxic cell fractions observed on FMISO PET (Figure 2C). These data are in partial agreement with Selvakumaran et al., where bevacizumab-treated HT-29/HCT116 xenograft tumours showed depletion of tumour microvasculature (i.e., anti-angiogenesis), and increased pimonidazole staining, consistent with an anti-angiogenic effect and induction of hypoxia in tumours [45]. In contrast, we found that HIF-1alpha was downregulated following cetuximab treatment in LIM1215 tumours (Figure 3), although both bevacizumab and cetuximab showed reduced hypoxic fractions on FMISO PET (Figure 2D), and both treatments reduced CD31 expression (Figure 3). These results indicate that cetuximab and bevacizumab also have anti-angiogenic effects which can result in alteration in vascular morphology and reduced hypoxia in tumours.

In summary, our results provide new insights into the interplay of signalling alterations and changes in metabolism and hypoxia response pathways in tumours following inhibition of VEGF and EGFR, and the inhibitory effects on both tumour cells and tumour microenvironment following treatment with cetuximab and bevacizumab. Most significantly, we demonstrate that these targeted therapies were selective on different tumour cells (resistant/weakly responsive), resulting in changes in tumour growth, metabolism, signalling, vasculature, and tissue oxygenation. Future studies which include gene knockdown and overexpression of proteins would provide further evidence of the mechanistic effects of changes in these identified altered proteins. These results identify biologic mechanisms underlying the anti-tumourigenic effects of cetuximab and bevacizumab in CRC, which may lead to a better understanding of the links between metabolism and tumourigenesis in cancer therapy and identifying response markers for anti-tumourigenic therapy.

**Table 4:** Hypoxia-related proteins identified in tumour xenografts in response to anti-tumour treatments

A - HT-29 cetuximab			HT-29^b^			LIM1215^b^		
Protein Acc^a^	Gene Name^a^	Protein Description^a^	Rsc (PBS v bevacizumab)	Rsc (PBS v cetuximab)	Rsc (PBS v Combination)	Rsc (PBS v bevacizumab)	Rsc (PBS v cetuximab)	Rsc (PBS v Combination)
P07996	THBS1	Thrombospondin-1	1.2	12.2	1.4	−15.6	−1.7	−14.2
Q9UJS0	SLC25A13	Calcium-binding mitochondrial carrier protein Aralar2	−1.5	3.0	−1.3	−2.0	−1.9	−1.9
Q06210	GFPT1	Glutamine--fructose-6-phosphate aminotransferase	−4.7	2.7	−4.1	−12.0	−10.9	−10.9
P55786	NPEPPS	Puromycin-sensitive aminopeptidase	−2.1	2.4	−1.8	−7.5	−2.0	−6.8
**B - HT-29 bevacizumab**								
O00151	PDLIM1	PDZ and LIM domain protein 1	9.1	1.1	4.9	2.3	−1.0	4.1
P07148	FABP1	Fatty acid-binding protein	7.1	1.1	8.3	−1.1	−1.0	−1.0
O14818	PSMA7	Proteasome subunit alpha type-7	6.2	1.1	9.5	5.8	−1.0	4.1
Q15370	TCEB2	Transcription elongation factor B polypeptide 2	6.2	1.1	1.4	3.0	−1.0	4.9
P42765	ACAA2	3-ketoacyl-CoA thiolase, mitochondrial	5.2	1.1	2.6	7.3	−1.0	7.2

a Protein description, Gene name, UniProt acc, and molecular function annotated from UniProt (http://www.uniprot.org/)

b Normalised spectral count ratio (Rsc) between datasets

## MATERIALS AND METHODS

### Cell lines

Human colon carcinoma LIM1215 cells [46] and human colorectal adenocarcinoma HT-29 cells [47] from ATCC were maintained in RPMI-1640 medium, supplemented with 10% foetal calf serum (RF-10), 10^−6^ M α-thioglycerol, 25 U/L insulin and 1 mg/mL hydrocortisone.

### Xenograft models

For xenograft and drug treatment assays, (5×10^6^ per site) LIM1215 or (2×10^6^ per site) HT-29 cells (5×10^6^ cells/site) were injected subcutaneously on Day 0 into BALB/c nude mice (Animal Research Centre, Perth, Australia) (*n* = 5) in left flank [48, 49]. The mice were maintained in microisolater cages housed in a positive pressure containment rack (Thoren Caging Systems Inc., Hazelton, PA). Food and water were provided in the cages. The mice were identified by earmarks and weighed twice a week. Tumours were measured twice a week using digital callipers. Tumour volume (TV) was calculated as (length × width^2^) / 2, where length was the longest axis and width was the perpendicular measurement [50]. Tumour volumes were expressed in mm^3^, and tumour growth curves were established over time for each cell line. The animals were euthanized when the TV reached 1000 mm^3^ or if there were any signs of distress prior to this.

### Therapy study

Treatment commenced when mean LIM1215 xenograft TV reached 208 mm^3^ and mean HT29 xenograft TV was 140 mm^3^. Groups of 5 mice for each xenograft model were randomly assigned to four treatment cohorts and treated for two weeks with, (1) Vehicle - 100uL PBS 2x/week; (2) cetuximab −20 mg/kg 2x/week; (3) bevacizumab −10 mg/kg 2x/week; or (4) the combination treatment both cetuximab (20 mg/kg) and bevacizumab (10 mg/kg) 2x/week.

During therapy mice were imaged with ^18^F-FDG and ^18^F-FMISO on a weekly basis before initial treatment, at commencement (week 0), at week 1 and at week 2. Tumour volumes were measured twice a week until mice were culled when either tumours reached >1000mm^3^ or if mice were deemed sick based on observation and/or weight loss. One mouse from each cohort was sacrificed when TV reached 200mm^3^, 500mm^3^, and 1000mm^3^, after imaging with each PET radiotracer, and correlative measurement of oxygenation was performed. For proteomic profiling and validation studies, tumours (*n* = 6) from three mice from each treatment cohort after 2 weeks were obtained, and tumours pooled in two separate groups (experimental and validation) and samples stored at −80°C.

The animal care and experimentation were performed according to the Australian Code of Practice for the Care and Use of Animals for Scientific Purposes endorsed by the National Health and Medical Research Council. The experimental protocols were approved by our institutional animal ethics committee.

### PET/MRI imaging

For the PET/MRI scans, the animals were anaesthetised in a halothane anaesthetic chamber to minimise background uptake of the radiotracer. The FDG PET scans were performed approximately 60 min after injection of 0.3mCi of ^18^F-FDG via a tail vein injection. The FMISO PET scans were performed 120 min after IV injection of 0.5mCi of ^18^F-FMISO. Food was withheld from the mice 2 h prior to FDG injection only, but not for FMISO imaging. The mice were injected with the relevant PET radiotracer prior to imaging.

All the PET/MRI imaging was performed on the Mediso nanoScan PM^®^ (Mediso Medical Imaging Systems, Budapest). The mice were in supine position with the head secured via ear and tooth bars. Respiration was monitored by a pressure-sensitive pad adhered to the abdomen. The MRI scan started with a whole body T1-weighted 3D imaging, followed by T2-weighted sequence over the tumour. Following PET list-mode acquisition, the scans were reconstructed using the Tera-TOMO^®^ reconstruction provided by Mediso. Subsequently, reconstructed PET and MR images were transferred to a research PACS system where the images were retrieved for processing by PMOD^®^ for VOI markup.

The mice were sacrificed by over inhalation of isoflurane, and then the tumours were dissected and embedded in Tissue Tek Optimal Cutting Temperature compound (Sakura Finetek, Torrance, CA), frozen in isopentone cooled in liquid nitrogen, and stored at −80°C, until further analysis.

### PET/MRI image analysis

The region of interest in each image (ROI) was defined on T2-weighted MRI, and transferred onto all PET images. For FDG PET, the Total Glycolytic Volume (TGV) which is the sum of all glycolytic activity in the tumour was calculated. For FMISO PET, the Total Hypoxic Volume (THV), which is the 3D sum of hypoxic uptake in the tumour, as well as the tumour to Normal Ratio (TNR) of FMISO uptake was also calculated.

### Protein extraction and quantification of tumour xenografts

For each treatment cohort pooled tumour xenograft samples (both experimental and validation) were thawed on ice. Total protein was harvested from three independent tumour samples in each experimental group (experimental and validation). For protein extraction, lysis buffer (5 mL of (4% (w/v) SDS, 20% (v/v) glycerol and 0.01% (v/v) bromophenol blue, 0.125 M Tris-HCl, pH 6.8)) with protease inhibitor cocktail (Complete, EDTA-free protease inhibitor cocktail, Roche) and 1 mM DTT was combined with homogenised tumours and sonicated for 180 s [49]. Homogenates in each experimental group were incubated at 95°C for 20 min and 60°C for 2 h. After centrifugation at 25,000*g* for 30 min, each supernatant was subjected to quantification, performed as previously described [29] based on 1D-SDS-PAGE / SYPRO^®^ Ruby protein staining densitometry. This quantification method is reproducible, has a linear quantitation range over three orders of magnitude [51], and is compatible with GeLC-MS/MS [29, 52].

### Preparation of tumour xenografts for immunohistochemistry

Four μm sections of the paraffin embedded tissue from different treatment groups were mounted onto SuperFrost® Plus slides (Menzel-Glaser, Braunschweig, Germany), deparaffinized and rehydrated prior to quenching of endogenous peroxidise using 3% H_2_0_2_ for 10min. Sections to be stained with GLUT-1 (Thermo Scientific, Waltham, MA, USA) or HIF1-alpha (Abcam, Cambridge, UK) were boiled in a 100°C citrate buffer water bath for 30 min prior to 90 min ambient temperature incubation with respective primary antibody. For CD31 (BD Pharmingen, San Jose, CA, USA), EGFR (Santa Cruz Biotechnology, Santa Cruz, CA, USA) and VEGF-A (Santa Cruz Biotechnology, Santa Cruz, CA, USA) staining, antibodies were applied to 5 μm frozen sections fixed in 4°C acetone prior to application of primary antibody. To allow visualization of the immunostaining, sections were incubated with the Envision anti-rabbit-HRP conjugated secondary antibody (Dako, Glostrup, Denmark) and DAB (Sigma, St. Louis MO, USA) and counterstained with Mayer's haematoxylin. Detection of CD31 antibody was done with Vectastain (Vector Laboratories, Burlingame, CA, USA) ABC anti-rat-biotinylated secondary antibody. The histologic appearance of the tissue sections stained for various samples were confirmed with H&E staining.

Stained sections were scanned using the Aperio (Leica Biosystems, Wetzlar, Germany) Scanscope XT and Aperio image analysis algorithms. For each section, the percentage of positive cells were scored for staining intensity as -, negative; +, weak; ++, moderate; and +++ strong, to give an overall H-score. CD31 was scored as positive pixels in areas of viable tumour and presented as a percentage of viable tumour area.

### SDS-PAGE

Samples were prepared in Lämmli sample buffer (0.06 M Tris-HCl, 2% (w/v) SDS, 10% (v/v) glycerol, 0.01% bromophenol blue, pH 6.8) containing 50 mM DTT and heated for 5 min at 95°C. Proteins were separated on a 4-12% NuPAGE^®^ Novex Bis-Tris Gel (Invitrogen) at 150V at constant current.

### Protein immunoblotting

For immunoblotting (10 μg), proteins were electrotransferred onto nitrocellulose membranes using the iBlot™ Dry Blotting System (Life Technologies) and membranes blocked with 5% (w/v) skim milk powder in Tris-buffered saline with 0.05% (v/v) Tween-20 (TTBS) for 1 h. Membranes were probed with primary antibodies [mouse anti-ATP5B (Santa Cruz Biotechnology; 1:200), mouse anti-GPD2 (Santa Cruz Biotechnology; 1:200), rabbit anti-ARF4 (Abcam; 1:1000), rabbit anti-SDC2/HSPG2 (OriGene; 1:500)] for 10 h in TTBS (50 mM Tris, pH 7, 150 mM NaCl, 0.05% (v/v Tween 20) at 4°C, followed by incubation with either IRDye 800 goat anti-mouse IgG or IRDye 700 goat anti-rabbit IgG (1:15000, LI-COR Biosciences). Fluorescent signals were detected using the Odyssey Infrared Imaging System, (v3.0, LI-COR Biosciences, Nebraska USA).

### Proteomic analysis

Proteomic experiments were performed in duplicate. Tumour xenograft lysates (20 μg) were separated using SDS-PAGE and proteins visualized by Imperial™ Protein Stain (Thermo Fisher Scientific). Each gel lane was cut into 17 individual gel bands (1-2 mm width) and individual gel slices destained (50 mM ammonium bicarbonate/acetonitrile), reduced (10 mM DTT (Calbiochem) for 30 min), alkylated (50 mM iodoacetic acid (Fluka) for 30 min) and trypsinized (0.2 μg trypsin (Promega Sequencing Grade) for 16 h at 37°C), as described [29, 53, 54]. RP-HPLC was performed on a nanoAcquity^®^ (C18) 150 × 0.15-mm i.d. reversed phase UPLC column (Waters), using an Agilent 1200 HPLC, coupled online to an LTQ-Orbitrap mass spectrometer equipped with a nanoelectrospray ion source (Thermo Fisher Scientific) [55]. The column was developed with a linear 60 min gradient with a flow rate of 0.8 μL/min at 45°C from 0-100% solvent B where solvent A was 0.1% (v/v) aqueous formic acid and solvent B was 0.1% (v/v) aqueous formic acid/60% acetonitrile. The mass spectrometer was operated in data-dependent mode and survey MS scans (300–1500 Th) acquired with the resolution set to a value of 30,000. Selected precursors were fragmented by CID and real time recalibration performed using a background ion from ambient air in the C-trap [56]. Up to five of selected target ions were dynamically excluded from further analysis for 3 min.

### Database searching and protein identification

Raw data were processed using Proteome Discoverer (v1.4.0.288, Thermo Fischer Scientific) and searched with Mascot (Matrix Science, London, UK; v1.4.0.288), Sequest (Thermo Fisher Scientific, San Jose, CA, v1.4.0.288), and X! Tandem (v2010.12.01.1) against a database of 125,803 ORFs (UniProtHuman, Feb-2015). Data was searched with a parent tolerance of 10 ppm, fragment tolerance of 0.6 Da, minimum peptide length 6, maximum peptide length 144, and max delta CN 0.05. Peptide lists were generated from a tryptic digestion with up to two missed cleavages, carbamidomethylation of cysteines as fixed modifications, and oxidation of methionines and protein N-terminal acetylation as variable modifications. Peptide spectral matches (PSM) were validated using Percolator based on q-values at a 1% false discovery rate (FDR) [29, 57]. With Proteome Discoverer, peptide identifications were grouped into proteins according to the law of parsimony and filtered to 1% FDR [58]. Scaffold (Proteome Software Inc., Portland, OR, v 4.3.4) was employed to validate MS/MS-based peptide and protein identifications from database searching. Initial peptide identifications were accepted if they could be established at greater than 95% probability as specified by the Peptide Prophet algorithm [58, 59]. Protein identifications were accepted, if they reached greater than 99% probability and contained at least 2 identified unique peptides. These identification criteria typically established < 1% false discovery rate based on a decoy database search strategy at the protein level. Proteins that contained similar peptides and could not be differentiated based on MS/MS analysis alone were grouped to satisfy the principles of parsimony. Contaminants, and reverse identification were excluded from further data analysis. UniProt was used to classify identified proteins based on their annotated function, subcellular localisation [60]. The Human Protein Atlas (www.proteinatlas.org) was used as an annotated resource to assess the tissue expression of proteins identified in this study [61].

### Semi-quantitative label-free spectral counting

Significant spectral count normalised (Nsc) and fold change ratios (Rsc) were determined as previously described [28, 29, 52, 54]. The relative abundance of a protein within a sample was estimated using Nsc, where for each individual protein, significant peptide MS/MS spectra (i.e., ion score greater than identity score) were summated, and normalised by the total number of significant MS/MS spectra identified in the sample. To compare relative protein abundance between samples the ratio of normalised spectral counts (Rsc) was estimated. Total number of spectra was only counted for significant peptides identified (Ion score ≥ Homology score). When Rsc is less than 1, the negative inverse value was used. The number of significant assigned spectra for each protein was used to determine whether protein abundances between HT-29 and LIM1215 cells, and different drug treatment categories (control, bevacizumab, cetuximab, combination). For each protein the Fisher's exact test was applied to significant assigned spectra. The resulting p-values were corrected for multiple testing using the Benjamini-Hochberg procedure [62].

### Statistical analysis

Student's *t*-tests (GraphPad v5.0) were calculated, with *p < 0.05 and **p < 0.01 considered statistically significant.

## SUPPLEMENTARY MATERIAL FIGURE AND TABLE





## Abbreviations

CRC: colorectal cancer
DMEM: Dulbecco's Modified Eagle's Medium
EGFR: epidermal growth factor receptor
FCS: fetal calf serum
mCRC: metastatic colorectal cancer
PET/MRI: positron emission tomography / magnetic resonance imaging
VEGF: vascular endothelial growth factor
