# Correction: Molecular profiling of cetuximab and bevacizumab treatment of colorectal tumours reveals perturbations in metabolic and hypoxic response pathways

**DOI:** 10.18632/oncotarget.9855

**Published:** 2016-06-06

**Authors:** David W. Greening, Sze Ting Lee, Hong Ji, Richard J. Simpson, Angela Rigopoulos, Carmel Murone, Catherine Fang, Sylvia Gong, Graeme O'Keefe, Andrew M. Scott

Present: Due to an error during manuscript preparation, loading control (GAPDH) was omitted from Figure 5.

Corrected: Correct Figure 5 is provided below. Authors sincerely apologize for this oversight.

Original article: Oncotarget. 2015; 6(35): 38166-80. doi: 10.18632/oncotarget.6241.

**Figure 5:**
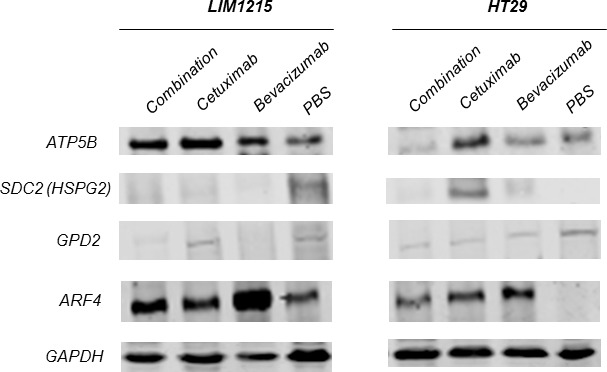
Validation of anti-tumour effects of cetuximab and bevacizumab mediated through altered cellular metabolism Proteins were extracted from tumour xenografts for each treatment cohort (independent from the tumour xenograft lysates performed for proteomic profiling), obtained from pooled tumour xenograft samples from the validation experimental group (n=3). Immunoblotting analysis of the expression of ATP5B, SDC2, GPD2, ARF4 in both LIM1215 and HT-29 tumour xenograft lysates was performed (n=3; pooled for each treatment cohort, independent biological replicates performed for each antibody). Loading control (GAPDH; rabbit anti-GAPDH (Cell Signaling Technology; 1:1000)).

