# Supplementary material for: Molecular profiling of cetuximab and bevacizumab treatment of colorectal tumours reveals perturbations in metabolic and hypoxic response pathways
Source: Oncotarget. 2015 Oct 26;6(35):38166–80. doi: 10.18632/oncotarget.6241 (PMC4741991; doi:10.18632/oncotarget.6241)
Supplement: Supplementary file 1 [file oncotarget-06-38166-s001.pdf]

Molecular profiling of cetuximab and bevacizumab treatment of colorectal tumours reveals perturbations in metabolic and hypoxic response pathways

Supplementary Material

A

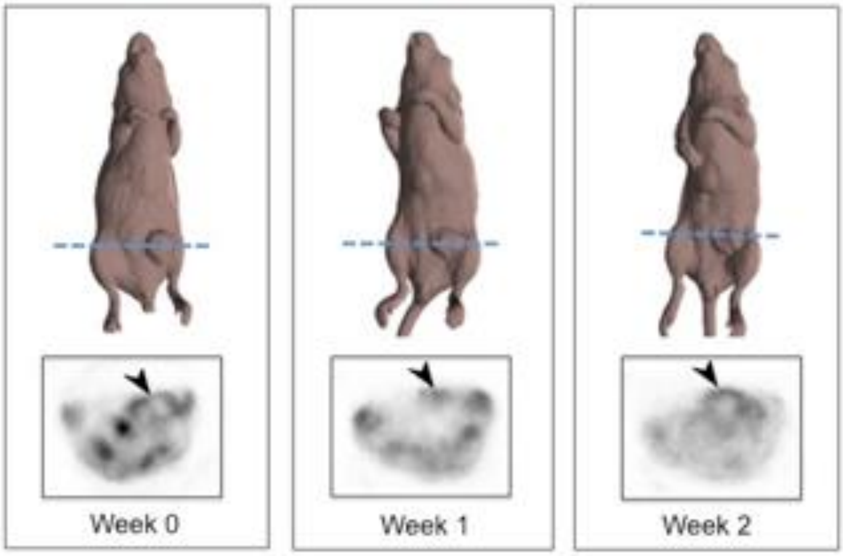

B

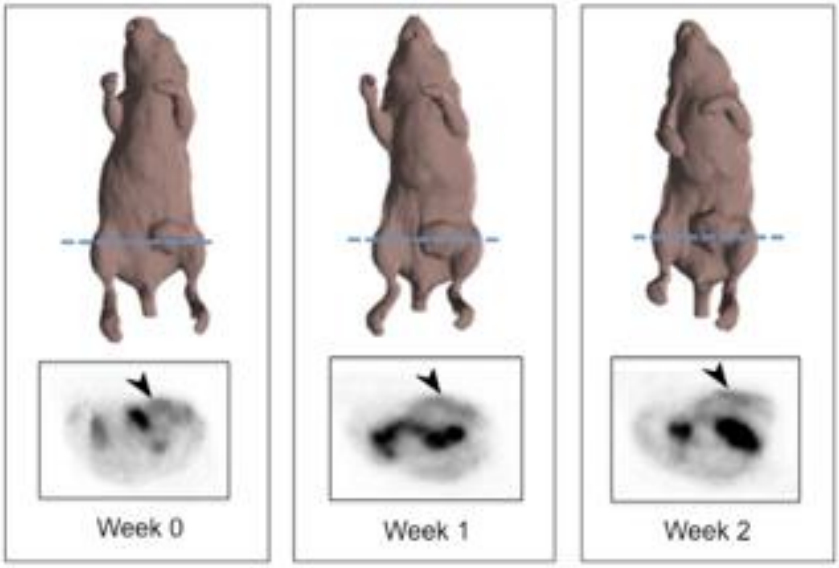

Supplementary Figure 1. Molecular imaging of metabolism and hypoxia in colorectal tumours after cetuximab treatment.

Whole body surface rendered MRI scans (top), and transaxial PET images (bottom) through tumour, are shown at baseline and following Cetuximab treatment. (A) ^{18}F-FDG PET and MRI images, and (B) ^{18}F-FMISO PET and MRI images. Arrows indicate site of tumour on transaxial PET images. Normal uptake of ^{18}F-FDG in muscle and bowel (A), and ^{18}F-FMISO excretion in bowel (B), is evident.

See Supplementary Table: Proteins identified in HT-29 and LIM1215 tumour lysates in response to therapeutic treatment

Spectral counts, and ratios (fold-change) of proteins in replicates of control (PBS), bevacizumab, cetuximab, and combination treatments of HT-29 and LIM1215 tumour lysates.
